# Lipoabdominoplasty: A versatile and safe technique for abdominal contouring

**Published:** 2008-10

**Authors:** Mohan Rangaswamy

**Affiliations:** Specialist Plastic Surgeon, Dubai, U.A.E

## INTRODUCTION

Abdominoplasty is an important and common operation in aesthetic surgery. Since its original recorded description, several technical improvements have been made. Fundamentally, however, there has been no change in the essential concept of extensive undermining almost to the costal margin, followed by appropriate resection and closure. The operation is still associated with a significant complication rate, morbidity and prolonged convalescence.[1–4] Complications have included flap necrosis, seroma, haematoma, infections, fat necrosis, dehiscence of wound and delayed healing. There is also a high incidence of aesthetic flaws and need for secondary correction, rates as high as 27.9 % being reported.[1] Extensive undermining causes denervation and reduction in the vascularity of the flap. There is thus an intrinsic design flaw in the traditional operation, namely, a large random pattern skin flap, partly deprived of blood supply and innervation is stretched maximally and sutured under tension.[3,4] This accounts for ischemia related complications. The lower abdominal skin also remains permanently numb.[5] The traditional technique also results in several lymphatics being divided. Despite routine use of drainage, a high rate of postoperative seroma is still accepted as unavoidable. Despite such high complication rate even the end results may not be always aesthetically pleasing.[6]

**Figure 1:**
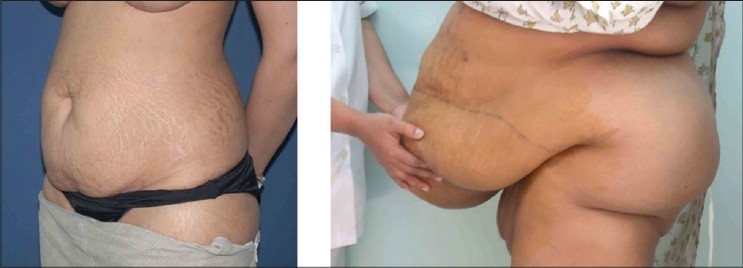
Left: An ideal patient for abdominoplasty. Right: the type of obese patient that one often gets

The problems increase significantly in obese subjects and hence patients are told to lose maximum weight in order to “earn” their abdominoplasty.[7] The best indication for abdominoplasty is still a patient with plenty of loose skin, rectus muscle diastasis and without too much fat in the local area. With the rising incidence of obesity in the world, such patients are rare in today's practice [Figure 1]. Many obese patients are unable to lose weight especially in the abdominal area and hence are denied abdominoplasty. In obese individuals unable to lose weight, a two step approach has been advocated consisting of abdominoplasty followed by liposuction six months later or vice versa. Both approaches are less than ideal. Reducing excess fat in abdominal wall by prior liposuction will induce fibrosis, making subsequent abdominoplasty difficult. Performing abdominoplasty first in these obese patients is associated with more complications and still leaves behind a fatty abdominal wall; subsequent liposuction can cause secondary skin laxity.

To circumvent these problems a new operation called Lipoabdominoplasty (LABP) has been practised by the author since 1995 with surprisingly low complication rate. Techniques have been refined over this period and in its present form it has proven to be trouble-free and versatile. Historically the concept of combining liposuction with abdominoplasty seems to have been proposed first by Cardoso de Castro in 1987[8] with later contributions by several surgeons.[9–12] However this procedure has not been adopted universally yet. Part of the reason for reluctance is the fear that combining the two operations may be risky as was suggested in earlier writings where the concept of “danger zones” was proposed.[12] Modern LABP is an entirely new operation and is not merely a combination of two procedures; this is important to understand in order to attain efficacy with safety.[13–16] In this article the author explores the philosophy of this new procedure, laying emphasis on the essential steps for safety to shorten the learning curve. Personal experience with a consecutive series of over 120 mostly obese patients is used to illustrate the points.

## PATIENT ASSESSMENT AND INCLUSION

### Consultation

During consultation due attention is paid to the following features. In the history it is important to elicit details of general fitness and functional capacity, lifestyle, smoking, co-morbidities, previous abdominal operations as well as evaluate risk profile for DVT and chest complications. Examination focuses on differentiating intra-abdominal obesity from obesity in the abdominal wall and the relative contribution of both components to the abdominal contour. This crucial factor decides if the obese patient is a candidate for LABP. Persons with predominant visceral obesity are counselled to lose weight and are not accepted for surgery. Further examination in suitable patients will focus on measuring the thickness of fatty panniculus, evaluating the degree of skin ptosis and vertical mobility of the abdominal panniculus, skin tone/elasticity, muscle tone, presence/absence of rectus diastasis/hernia [Figure 2a], previous surgical scars including laparoscopy scars, presence and extent of skin striae, the position of umbilicus vis-á-vis the pubis and presence/absence of ptosis of mons pubis. An assessment is then made of the extent of dermolipectomy possible in order to forecast if the umbilical donor opening can be excised completely. The possibility of inverted T closure will have to be explained to the patients with lesser deformities. Other areas such as back, hips, and thighs are also assessed and the respective body contour problems discussed with the patients to clearly set out the goals of LABP and to indicate the need for other body contour procedures that may be needed in each case. This is especially important since patients with iliac and hip fat rolls will end up with lateral bulges if these areas are not addressed. Patients often complain bitterly about such bulges unless this is discussed and addressed either simultaneously or in a planned second body contour procedure.

**Figure 2a:**
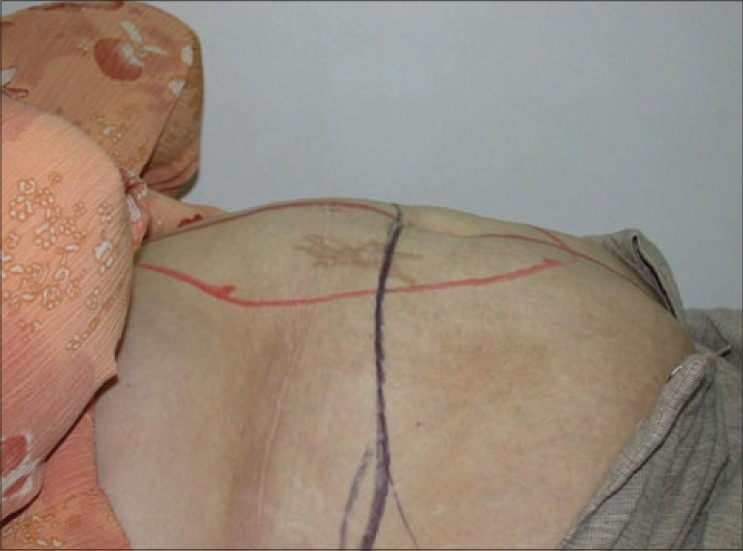
Preoperative view, patient in supine position. Red marking shows the extent of rectus muscle diastasis and black line is the proposed upper limit of skin resection. Same case as in fig 4

### Further points for decision making in obese individuals

Obesity comes in different shapes; Body Mass Index (BMI) is an inadequate tool to decide on the inclusion for surgery. In a significant proportion of obese females the fat is predominantly in the abdominal wall. Such obese patients can be accepted for surgery provided a useful degree of improvement can be anticipated after surgery and provided that such improvement fits into the overall scheme of lifestyle changes and medical management. Decision making is easy in patients classified as overweight or with obesity grade I and II especially if the problem is more significant in the abdominal wall. Decision has to be individualized in obesity grade III and in the morbidly obese. In these the individual distribution of obesity in the body, functional status, motivation and lifestyle factors and repeated discussions may be needed to arrive at a decision. In male patients and in android female patients a very careful assessment of the sagittal abdominal diameter must be made to exclude patients whose apparent abdominal contour is due to visceral fat [therefore not correctable by surgery].

### Contraindications

Patients who are medically at high risk like those with uncorrected coagulopathies, significant cardio-pulmonary compromise and viscerally obese patients, globally obese and functionally compromised patients and patients with unrealistic expectations are to be excluded. Smokers who cannot give up the habit need to be counselled carefully regarding risks and potential complications which tend to be higher in them.

### Preoperative routine

The author has been using the following measures.

1. Complete medical evaluation and optimisation by a physician in patients with co-morbidities
2. A month of regular aerobic exercises in sedentary individuals to build up cardio-respiratory reserves and to unmask latent functional problems.
3. Full body scrub with Povidone iodine or Chlorhexidine preparations for 3 days prior to surgery, paying attention to intertriginous areas and umbilicus
4. Pre-anaesthesia evaluation and appropriate corrections where needed
5. Attention to diet
6. Routine use of low-molecular weight Heparins and anti-embolism stockings.
7. Measurements for postoperative garment based on hip size excluding the apron.
8. Markings are done on the previous day with the patient upright to mark the midline, xiphoid and pubic symphysis, proposed incision and estimated dermolipectomy. Any hernia sites are marked and the diastasis is similarly outlined.

### Surgical procedure

#### Positioning and preparation

Patient is positioned supine on the table with a removable bolster under the lumbar lordosis to hyper-extend the abdominal wall. Catheterization is done if procedure is anticipated to exceed 150 minutes. Pressure points are padded and pneumatic calf compression pump is applied in high risk individuals. General endotracheal anaesthesia is induced. Draping is done widely with a sterile sheet tucked under the flank to give full flank access for liposuction. Drapes are placed above the xiphoid and below the pubis for adequate access. Prophylactic broad spectrum antibiotic of choice is administered; the author prefers 2g of Cefipime IV after induction of anaesthesia.

#### Liposuction

Procedure begins with infiltration of tumescent fluid (Klein formula) in all areas to render the tissues firm but not completely tumescent.[17] Liposuction is done using standard technique by the surgeon's preferred method. I do not recommend cannulae larger than 4 mm. Mercedes three hole cannulae are preferred. More aggressive cannulae can damage vasculature and are not recommended. Suction is done uniformly in all areas, there are no danger zones. Care is taken within the area of diastasis of recti and near hernias to avoid peritoneal entry; the canula is always kept tangential to the abdominal dome. Since the lower panniculus will be excised, only deep plane suction is done there for lipomobilization effect. The midline and paramedian upper abdomen is suctioned more vigorously to reduce the fat thickness to 1 cm pinch thickness, more fat is retained on periumbilical area and along linea semilunaris [1.5 cm pinch thickness]. Flanks and hips can be suctioned aggressively to sculpt the waist. Suction is also done below the final incision and into mons pubis as needed. Scarpa's fascia is not violated. Generally liposuction may be continued till 1 cm pinch thickness is reached as long as care is taken to avoid aggressive scraping of the skin. The purpose of the exercise is to thin the subcutaneous fat and get maximum lipomobilization effect to enable the upper abdominal skin to slide inferiorly while retaining vascularity *via* the numerous perforators. Quantity of fat suctioned from abdomen alone has ranged from 50 ml to 4000 ml in my cases.

#### Abdominoplasty

After liposuction, the lumbar bolster is removed and the lower incision is made. Incision placement is a crucial step. In women with sufficient ptosis of the skin, the incision is placed in the abdominal crease whenever possible respecting the guidelines laid out by Baroudi and Moraes.[18] Baroudi laid out a bicycle handle-bar type of low incision which enables the surgeon to resect more skin in the flanks and keep the final scar low and aesthetically pleasing. The flank line is tightened well while also ensuring a youthful separation of the umbilicus from the pubis. In women with less ptosis, it helps to keep the central part of the lower incision convex cranially to avoid tension in the midline at the time of closure. The gives the crucial 2 cm of extra lower skin to enable tension free closure at the midline. If not needed at the end, this extra skin can be trimmed. The final closure should be 7-9 cm from the top of vulval commissure for aesthetic result and patient comfort. The aesthetics of the pubic region must also be maintained, avoiding the distressing upward migration of the pubic hair triangle as well the pulled appearance of the vulva. In women with ptosis of the mons pubis, a mons-lift is planned and factored into the incision placement.

Using cutting diathermy, incision is deepened to incise Scarpa's fascia but no deeper. The plane of dissection then turns cephalad, closely hugging the under-surface of Scarpa's fascia. The adipose tissue on the external oblique aponeurosis is left intact; this has been shown to be rich in lymphatics.[14] Any violation of these tissues increases lymphorrhea and results in seroma. Flap elevation is continued to the level of umbilicus. Sharp dissection stops at this level. Beyond this the honeycombed under surface of the flap and intact fibro vascular connections can be visualised [Figure 2b]. The umbilicus is circumscribed and the stalk dissected with a thin layer of fat on it. Some peri-umbilical fat is also retained on the rectus fascia. Rectus perforators are pre identified and securely tied [not merely cauterized]. These perforators are in spasm due to the effect of adrenaline and can slip inward, causing rectus sheath haematoma. Supra-umbilical perforators are preserved as far as possible. Flap elevation then proceeds above umbilicus but only in the midline and paramedian areas to expose the rectus sheath for an inch beyond the respective medial borders [stretched linea alba]. Midline plication is done only if there is diastasis; plication is continued inferiorly till the pubis. I routinely use PDS loop suture but have recently switched over to No 2 Ethibond® which is a soft non-absorbable permanent suture. The operating table is then flexed about 30 degrees and a trial closure is done to assess the extent of dermolipectomy possible. Further selective blunt digital flap elevation is performed going between the intact fibrovascular connections to mobilise the sliding flap further. An occasional fibrous strand may need to be divided sharply. Maximal dermolipectomy is then performed mirroring the pattern of the lower incision; it is important to plan this carefully to avoid excessive tension on closure, it is better to err on the side of safety. Further trimming can always be done where needed. Closure is tested with a few trial sutures.

**Figure 2b:**
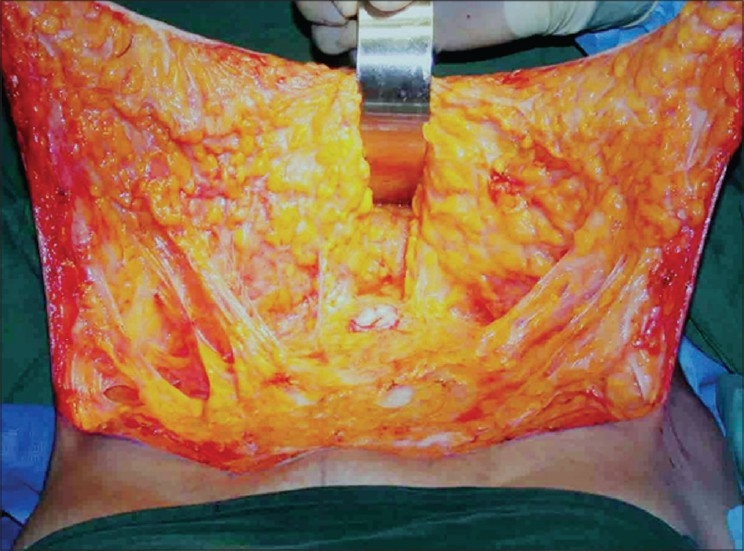
Inner surface of lipomobilized flap after selective undermining. Note the umbilicus in the centre and the retractor cranial to that. Midline plication is easily possible. Note the long strands of fibro-neuro-vascular connections and also the intact layer of adipose tissue left behind on the external oblique aponeurosis. This tissue is rich in lymphatics

#### Umbilicoplasty

The umbilical stalk is telescoped with four quadrant sutures tacking it to the rectus sheath. This step is important to give a proper inversion to the future umbilicus. The umbilical location is projected on the skin flap and marked with ink. The new umbilical opening is planned 1.5 to 2 cm cranial to the upper border of the projected point. The new site is de-epithelialised and the dermis incised in tri-radiate fashion. A divergent cone of fat is then removed beneath this area. This step is important for a nice concave umbilicus. Two strong vicryl sutures are taken from the dermal fringes at 3 and 9 ‘o’ clock positions and anchored to the rectus fascia on either side of the umbilical stalk. This important step further advances the abdominal flap downwards and puts higher tension on the upper part where the flap vascularity is better while simultaneously relaxing tension at the lower closure. It also achieves an ink-well effect of a concave umbilicus. A suture may also be taken at 12 ‘o’ clock position if needed.

After this, the dermal fringes of the umbilical opening are sutured to the umbilical stalk to take the tension off the final skin closure which is done with interrupted 4-O *rapide* Vicryl®.

#### Obliterating the dead space

The skin flap is advanced and the dead space is obliterated with 4 to 8 quilting sutures per side of 2-O Vicryl *rapide*® taking bites from the dermis to the external oblique fascia and going through the iliac adipolymphatic pads. Drains are not necessary. The incision is closed in 3 layers compensating the superior [longer] flap medially to correct any length disparity. It is important to close the superficial condensed fascia properly taking all the tension there and avoiding any tension on the skin closure.

### Postoperative regime

After extubation and postoperative recovery, the patient is assisted into the appropriate postoperative compression garment.

The patient is nursed in the Fowler position i.e. with head end of bed propped up and a pillow under the knees to flex the hips. Urinary catheter, if any, is retained for a day. Patients are routinely ambulated the same day and mostly discharged the next day. In cases of significant midline plication or hernia repair, patients stay for 2 days. Low molecular Heparin is continued the next day, stockings are worn for 5 days and the first visit is also on the 5^th^ day. Compression garment is worn for 3 weeks and strenuous exercises are avoided. Guided exercises are begun thereafter.

### Summary of results of the author's series

The author has used the described technique in 135 consecutive cases, 120 of which have been analysed for publication. Most of the patients were females (112). 90% were either clearly obese or overweight (mean BMI 33), the heaviest weighed 143Kg. Thirty seven patients also had co-morbidities like type 2 diabetes, hypertension and chronic airway disease. 19% were smokers. Forty three had previous abdominal surgeries (36%) including 8 upper midline scars and 5 transverse scars. 27 had associated true hernias and 32 had rectus muscle diastasis. Liposuction volumes averaged 953 ml and lipocrit ranged from 50-4200 ml. Volumes were over 1000 ml in a third of the patients. The excised tissue weight ranged from 50g to 12Kg (mean 1596g). 52 additional procedures were also performed simultaneously with LABP including 13 laparotomies. There were 20 complications in 18 patients in the entire series (16.6%) including all minor issues. The rate was 23% in the first 30 cases and 13% in the last 30 (4/30 namely lower lung atelectasis, acute gastric dilatation (both patients also underwent simultaneous hysterectomy), umbilical ischemia in a case with large recurrent ventral/umbilical hernia and one hematoma in an Aspirin user which required aspiration). On further analysis, only 4.8% of complications were directly attributable to the procedure. Significantly, the last 100 cases had no seromas, only 2 hematomas and no wound infections or significant delays in healing. There were no major wound problems or infections; two required secondary liposuction for lower abdominal contour improvement. None of the smokers developed wound problems.

Patient outcomes were graded on a 4 point scale from poor to excellent based on whether there were major complications requiring further surgery for salvage (poor) or minor interventions early in the course for recovery (fair) or only aesthetic dissatisfaction requiring later interventions such as liposuction or dog-ear correction (good) or no interventions (excellent). In the series of 120 cases, the result was excellent in 93 cases [Figures 3, 4], good in 19 and fair in 8 cases. There were no poor outcomes.

**Figure 3a:**
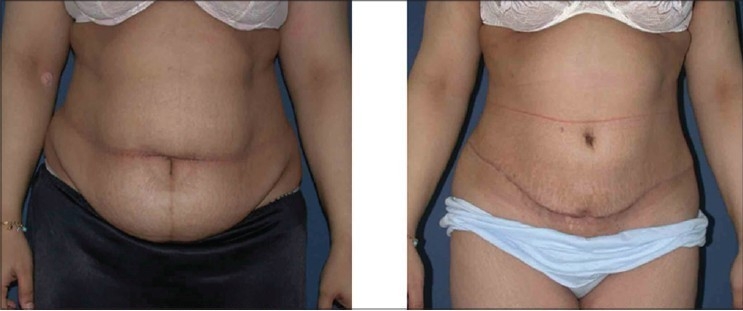
Upper row: Front views, Left: Pre-operative Right: 12 weeks postoperative

**Figure 3b:**
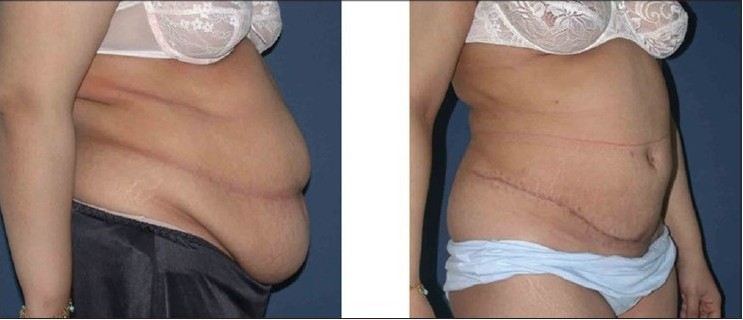
Middle row: Side views, Left: Pre-operative; note obesity of abdominal wall and tissue laxity. Right: 12 weeks post-operative

**Figure 3c:**
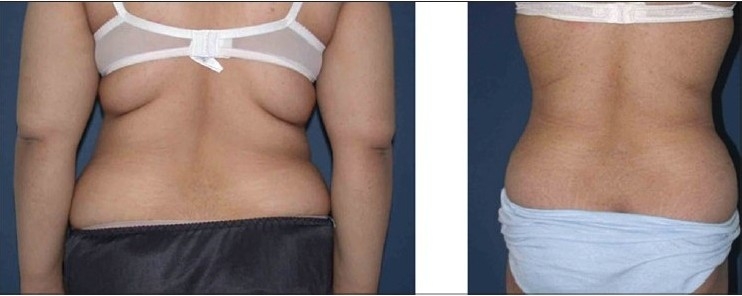
Lower row: Back views, Left: Pre-operative view. Right: 12 weeks post-operative view after circumferential liposuction and LABP

**Figure 4a:**
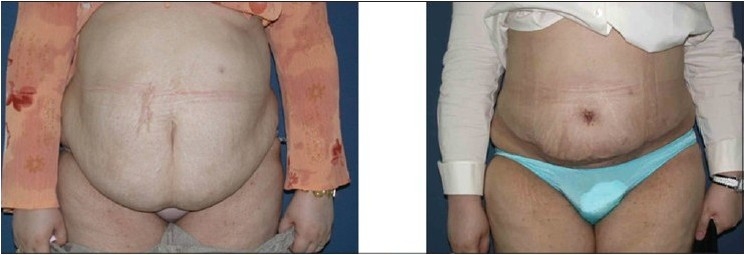
Upper row: Front views, Left: Pre-operative, note the abdomina apron hanging in front of the mons pubis and the extent of obesity. Right: 10 weeks post-operative

**Figure 4b:**
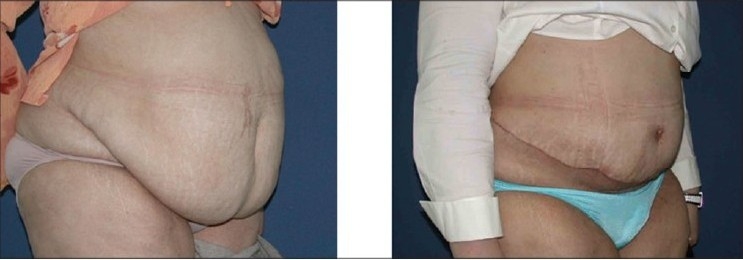
Lower row: Oblique views: Left: Pre-operative. Right: 10 weeks post-operative

## Discussion

Use of liposuction as an adjunct to abdominoplasty is not new.[8–12,15] It was probably first suggested by Castro *et al*. in 1987[8] and repeatedly by various surgeons particularly Illuoz,[9] Dillerud,[10] Matarasso.[12] Matarasso coined the term abdomino *lipoplasty* to describe this more accurately. Initial attempts however ranged on one extreme from timid addition of liposuction to some areas avoiding “danger Zones” to the other extreme of adding only lower abdominal dermolipectomy and midline placation to extensive liposuction.[9,12,19] Combining full abdominoplasty with extensive liposuction was and is considered risky. This is because the abdominoplasty was still performed in the traditional way with full undermining. Older studies also used larger cannulae in liposuction. There were however publications appearing periodically advocating the combination of both modalities and attesting to the safety.[11,15,16]

Lipoabdominoplasty described in this article in its present form is a new operation. The author has been performing this combination surgery since 1995 and particularly so since 2000 incorporating newer advances. Evolution of the technique has followed contributions from several surgeons, some of whom are credited in the bibiliography especially the work by LeLouarn *et al*. and by Saldanha *et al*.[13–16] It is not a mini abdominoplasty with liposuction but is versatile enough to address Matarasso types 2 to 4 deformities.[12] (Matarasso classified abdominal deformities for surgical correction into 4 types. Briefly: type 1 patients have minimal or no laxity of skin or deeper tissues and can be managed by liposuction alone, type 2 have mild laxity in lower abdomen and need a mini-abdominoplasty, type 3 have moderate flaccidity of lower or upper or both parts of the abdominal wall and need a modified abdominoplasty where the umbilical hole has to be closed separately after umbilical transposition and finally type 4 where there is gross abdominal flaccidity requiring full abdominoplasty).

The important principles of LABP are repeated here. Safety and success is based on respecting these principles. The author has steadily extended the indications to include even patients with obesity grade 3 and some morbidly obese patients with very fatty abdomens with surprisingly safe outcomes. This attests to the validity of the principles. LABP has also been combined with other body contouring procedures for 3-D contouring as well as with other facial or breast aesthetic procedures.

LABP starts with extensive liposuction first to reduce the fat in the abdominal panniculus and to achieve lipo-mobilization of the flap. Only 3 and 4 mm cannulae are used by the author. Liposuction first has several advantages in all patients and especially in the obese. In obese patients, the flap in traditional abdominoplasty remains fatty and thick. This fat is a parasite on the blood supply of the flap and is susceptible to fat necrosis, with all the resultant sequalae of infection and contour irregularities. In LABP, liposuction eliminates most of the parasitic fat but retains many neurovascular connections intact.[20] In fact, thicker the abdominal wall fat, better is the mobilization achieved. The thinned abdominal flap also can reveal anatomical contours and musculature better avoiding the featureless flat abdomen look.

After liposuction the skin flap becomes a sliding flap based on a type of “tree-top” mobility. Surgical undermining in most cases is required only up to the level of umbilicus. After such selective undermining, the flap is able to reach down well to allow the requisite skin excision and closure. Concerns about the vascularity of such a liposuctioned flap are unfounded. In fact robust blood supply is routinely observed at surgery and this is further attested by zero necrosis in the last 100 consecutive cases in the author's series. Peri-umbilical perforators fed by the epigastric vascular axis are retained intact as much as possible especially the supraumbilical ones. These perforators are an important source of nutrition to the abdominal wall forming the basis of TRAM flap and several umbilical perforator based flaps. Such flaps have also been raised and used successfully from abdomens subjected to prior liposuction attesting to the fact that vasculature is preserved after liposuction.[20] A study by Graf R *et al*, confirmed intact perforators after Lipoabdominoplasty.[21] The other advantage of prior liposuction is that blood loss is drastically reduced to negligible limits due to the effect of the wetting fluid infiltration. Further, tunnelled midline undermining allows plication of the midline muscles as needed; if necessary all the way from epigastrium to pubis.

The second modification of raising the abdominal flap strictly on the undersurface of Scarpa's fascia avoids cutting the important lymphatics beneath and this is turn reduces drastically the incidence of seroma. Abdominal flap oedema is also minimized.[14]

The third modification of siting the new umbilicus 1-2 cm cranial to the upper border of the isolated umbilicus creates strong tension in the upper [more vascular] part of the flap and relaxes the tension on the lower [less vascular] part of the flap. There are additional benefits of this step:

1. It ensures a concave “dip” of the flap around the future umbilicus by nailing the flap to the rectus fascia to simulate the natural anatomy of the umbilicus.
2. It flattens the upper abdomen.
3. Relieves any tension on the umbilical closure and thus future spreading of the umbilical scar.
4. Gives sufficient separation between the umbilicus and the pubis for an aesthetically youthful abdomen.

The fourth important refinement is the deliberate obliteration of the dead space in the infra-umbilical area by quilting sutures. These serve to progressively advance the flap, distribute tension and most importantly prevent seroma by eliminating dead space.[14,22] These sutures help to subdivide the dead space into smaller spaces while acting also as additional immobilization points thereby eliminating seroma as well as reducing tension on the final closure. Tension is a well known enemy of flap vascularity. The upper abdomen has no dead space as it is not undermined, only lipomobilized, hence quilting is not needed there. Eliminating drains takes away one source of patient discomfort and allows early discharge from the hospital. The author has not used drains in the last 115 consecutive cases with zero incidence of seroma.

Finally a point about wound closure. It is important to close the deep condensed fascia of the upper edge to the remnant of Scarpa's fascia on the lower edge by strong sutures to achieve a good contour as well as to relieve tension on the skin layer (there is no Scarpa fascia on the upper edge as it is supra umbilical skin). This translates into superior scar quality.

## Conclusion

Lipoabdominoplasty is a new, more physiological and versatile operation that drastically reduces complications, extends the indications for patient recruitment to the obese and super obese populations and simplifies the surgery while also giving a better aesthetic result with quicker recovery. The extremely low incidence (see results) of complications in the author's series of potentially high risk patients when contrasted with the high complications reported in literature about traditional abdominoplasty carries an important message about the safety of this operation. In the author's opinion the good results are the direct consequence of the more physiological design of Lipoabdominoplasty. Similar good results have been reported by other workers as well.[13,14,21] It is not indicated in the rare patients with very thin abdominal walls.
